# Draper’s Physiology

**Published:** 1856-10

**Authors:** 


We have before us a copy of Draper's Physiology, published by Harper & Bros. Our time and space in this No. of the Journal, will not admit of such an extended notice as this important work demands. We will allow ourselves the pleasure of giving this excellent production such attention as it deserves.

